# Machine Learning Prediction of Treatment Response to Inhaled Corticosteroids in Asthma

**DOI:** 10.3390/jpm14030246

**Published:** 2024-02-25

**Authors:** Mei-Sing Ong, Joanne E. Sordillo, Amber Dahlin, Michael McGeachie, Kelan Tantisira, Alberta L. Wang, Jessica Lasky-Su, Murray Brilliant, Terrie Kitchner, Dan M. Roden, Scott T. Weiss, Ann Chen Wu

**Affiliations:** 1 PRecisiOn Medicine Translational Research (PROMoTeR) Center, Department of Population Medicine, Harvard Medical School and Harvard Pilgrim Health Care, Boston, MA 02215, USA; joanne.sordillo@va.gov (J.E.S.); annchenwu@alum.mit.edu (A.C.W.); 2 Channing Division of Network Medicine, Brigham and Women’s Hospital and Harvard Medical School, Boston, MA 02115, USA; readh@channing.harvard.edu (A.D.); remmg@channing.harvard.edu (M.M.); reawa@channing.harvard.edu (A.L.W.); jessica.su@channing.harvard.edu (J.L.-S.); restw@channing.harvard.edu (S.T.W.); 3 Division of Pediatric Respiratory Medicine, Department of Pediatrics, University of California San Diego and Rady Children’s Hospital, San Diego, CA 92123, USA; ktantisira@health.ucsd.edu; 4 Marshfield Clinic Research Institute, Marshfield, WI 54449, USA; brilliant.murray@mcrf.mfldclin.edu (M.B.); kitchner.terrie@mcrf.mfldclin.edu (T.K.); 5 Department of Medicine, Vanderbilt University Medical Center, Nashville, TN 37232, USA; dan.roden@vumc.org

**Keywords:** asthma, inhaled corticosteroids, polygenic risk prediction, machine learning, pharmacogenetics

## Abstract

Background: Although inhaled corticosteroids (ICS) are the first-line therapy for patients with persistent asthma, many patients continue to have exacerbations. We developed machine learning models to predict the ICS response in patients with asthma. Methods: The subjects included asthma patients of European ancestry (*n* = 1371; 448 children; 916 adults). A genome-wide association study was performed to identify the SNPs associated with ICS response. Using the SNPs identified, two machine learning models were developed to predict ICS response: (1) least absolute shrinkage and selection operator (LASSO) regression and (2) random forest. Results: The LASSO regression model achieved an AUC of 0.71 (95% CI 0.67–0.76; sensitivity: 0.57; specificity: 0.75) in an independent test cohort, and the random forest model achieved an AUC of 0.74 (95% CI 0.70–0.78; sensitivity: 0.70; specificity: 0.68). The genes contributing to the prediction of ICS response included those associated with ICS responses in asthma (*TPSAB1, FBXL16*), asthma symptoms and severity (*ABCA7, CNN2, PTRN3,* and *BSG/CD147*), airway remodeling (*ELANE, FSTL3*), mucin production (*GAL3ST*), leukotriene synthesis (*GPX4*), allergic asthma (*ZFPM1, SBNO2*), and others. Conclusions: An accurate risk prediction of ICS response can be obtained using machine learning methods, with the potential to inform personalized treatment decisions. Further studies are needed to examine if the integration of richer phenotype data could improve risk prediction.

## 1. Introduction

Inhaled corticosteroids (ICS) are the most commonly used controller medications for asthma, which affects over 300 million people worldwide [1]. However, a significant proportion of subjects continue to have exacerbations despite therapy [2,3]. Up to 55% of children with persistent asthma may not respond to ICS during an 8-week therapy course [4]. Moreover, the prolonged use of ICS exposes patients to adverse systemic effects, including decreased bone mineral density, cataracts, and adrenal suppression [5,6].

There is increasing evidence indicating that genetic variation substantially influences ICS efficacy [7]. Previously identified single nucleotide polymorphisms (SNPs) associated with ICS response include variants in *FCεR2*, *ST13, IL1RL1, CRHR1,* and *TBXT* [8,9,10,11,12,13]. Genomic studies using RNA-Seq to characterize transcriptomes have also identified multiple genes involved in the inflammatory pathway that influence ICS response. For example, *CRISPLD2* mRNA has been shown to increase in response to treatment with a pro-inflammatory cytokine (IL1β) [14]. Despite these early discoveries, most genetic variants associated with ICS response confer small-to-modest effects and cannot, by themselves, accurately predict ICS response in individual patients. Many complex traits are highly polygenic, whereby multiple causal variants simultaneously contribute to the genetic susceptibility of a trait [15]. Thus, although the risk conferred by individual SNPs may not be sufficiently large to predict a trait, the combined effect of multiple variants can achieve a degree of risk discrimination that is useful for risk assessment. However, developing the optimal methods for selecting and combining SNPs for risk prediction remains a significant question.

The objective of this study was to develop machine learning models for predicting ICS response using genome-wide genotype data from multiple cohorts of individuals with asthma. We developed and compared two machine learning models to predict the ICS response in patients with asthma: LASSO regression and random forest (a non-linear model). To date, no published studies have evaluated the use of machine learning models to predict ICS response in asthma robustly for multiple cohorts. The ability to stratify individuals based on their likely treatment response will offer the potential to optimize asthma treatment and to prevent treatment-related adverse effects.

## 2. Materials and Methods

### 2.1. Study Subjects and Datasets

This study’s cohort comprised 1371 asthma subjects of European ancestry with a history of chronic ICS use from seven well-characterized asthma cohorts with genome-wide genotype data. The pediatric asthma population included ICS treatment arms within the Childhood Asthma Management Program (CAMP) [16], and two of the five trials in the Childhood Asthma Research and Education (CARE) network—the Pediatric Asthma Controller Trial (PACT) and the Characterizing Response to Leukotriene Receptor Antagonist and Inhaled Corticosteroid (CLIC) trials [17,18]. The adult asthma cohort comprised subjects from the Asthma Clinical Research Network (ACRN), and data from two biorepositories linked to deidentified electronic health records from the PharmacoGenomic discovery and replication in very large POPulations (PGPop) cohorts: the Marshfield Clinic Personalized Medicine Research Project (PMRP) [19] and Vanderbilt University Medical Center’s BioVu program (BioVu) [20]. CAMP, CARE, and ACRN are part of the Single-Nucleotide Polymorphism Health Association-Asthma Resource Project (SHARP)—a data resource funded by the NHLBI that compiles genome-wide SNP data, along with clinical drug-treatment response data, from a large number of NHLBI-sponsored asthma clinical trials [21,22,23,24,25,26]. PGPop is a collaborative research resource of the Pharmacogenomics Research Network (PGRN). The institutions that are part of PGPop investigate drug-response phenotypes through the genetic testing of EHR-linked biobank data [21]. The combined datasets provide the larger sample size required for GWAS analyses and have been used in many published GWAS studies [22,23,24,25,26,27,28]. Supplementary Table S1 describes the population captured in each dataset. The subjects who were present in more than one study population were removed prior to evaluation. All study procedures were approved by the respective Institutional Review Boards of each consortium and the Brigham and Women’s Hospital (the Partners Human Research Committee (PHRC)). Human subjects approval was obtained from the Partners Human Research Internal Review Board, Protocol #: 2002P000331. Written informed consent was obtained.

From a total sample size of 1371 subjects, we randomly selected 823 subjects as the training cohort for model development, and the remaining 548 subjects as the test cohort for model validation.

### 2.2. Study Outcomes

The primary outcome was asthma exacerbations occurring while having two or more fills of ICS in a year, since two or more yearly fills of ICS is associated with a good treatment response and a significant decrease in hospitalizations from asthma [29]. An asthma exacerbation was defined as an emergency department (ED) visit or hospitalization due to asthma, or the need for oral corticosteroids. The high morbidity associated with these outcomes drives our focus on these areas.

### 2.3. Genotyping, Imputation, and Quality Control Procedures

Genotyping of DNA samples from the subjects enrolled in the six study populations has been previously described [4,16,19,20,30,31]. To account for the differences in the genotyping arrays and platforms used in each individual study, genetic markers across all five populations were merged using PLINK v.1.9 [32], pre-phased using Shape-IT v2.5 [33], and imputed to the 1000 Genomes Project (phase 1 integrated release [34]) reference CEU panels with IMPUTE2 [35].

Standard quality control procedures were applied to the merged, imputed dataset using PLINK v.1.9 to remove markers with below-threshold genotype call rates (<5%), low minor allele frequency (<5%), and Hardy–Weinberg Equilibrium deviation (*p* < 1 × 10^−6^). Principal components analysis was performed using PLINKv1.9 to adjust for population stratification. A final dataset of 5,401,598 variants and 1371 subjects passed all the filters and quality control measures for analysis.

### 2.4. Development of Predictive Models

To select the SNPs for inclusion in the predictive models, we first conducted a genome-wide association study (GWAS) on the training cohort to identify the SNPs associated with ICS response. The analysis was adjusted by the first 6 principal components. Linkage-disequilibrium–independent associations were obtained by clumping with an r^2^ threshold of 0.50, a physical distance of 250 kb, a significance threshold of 1 × 10^−5^ for the index SNPs, and a secondary significance threshold of 1 × 10^−2^ for the clumped SNPs. Since the goal of this pre-selection step was to reduce the dimensionality of the predictors to a manageable set, a less stringent GWAS threshold was applied to select SNPs. Using the selected SNPs, we then developed and compared two machine learning models to predict ICS response: (1) LASSO (least absolute shrinkage and selection operator) regression models and (2) random forest models. LASSO is an extension of ordinary least squares regression that performs both variable selection and regularization to enhance prediction accuracy [36]. Random forest is a classifier consisting of a collection of tree-structured classifiers, where the classifiers are independent, identically distributed random vectors, and each tree casts a unit vote for the most popular class [37]. Both modeling approaches involved performing further feature selection while fitting the predictive model. In the LASSO regression model, the number of SNPs entered into the final model depended on the LASSO regularization term. To identify the most optimal model, we fit multiple models using varying values for the LASSO regularization term, and evaluated them using balanced bootstrap resampling (with 100 iterations) on the training cohort. The final model was then validated on the hold-out test cohort. A similar process was conducted to fine-tune and optimize the random forest model, whereby multiple models were developed with a varying number of variables randomly sampled as candidates at each split, and the most optimal model was selected. In both approaches, the models were optimized to maximize the area under the receiver operating characteristic curve (AUC) on the training data. The SNPs that contributed the most to the prediction of ICS response in each model were identified using the measure of variable importance—a ranked variable ranging from 0 to 100 that quantifies the importance of each variable in the prediction models.

To determine whether the combination of genetic and phenotype data more accurately predicts ICS response than genetic data alone, we further developed separate models integrating SNPs and phenotype data (including sex, age, and body mass index [BMI]) to predict ICS response. In a traditional GWAS analysis, variability in the phenotypic characteristics can confound the relationship between genetic variants and the outcome of interest. The most common approach with which to address confounding effects is by regressing the covariate on the genetic variant. However, because our goal was to predict ICS response and not to identify the effect of a particular variant on ICS response, phenotype data were included as potential predictors in the machine learning models instead. This allowed for the machine learning models to learn any complex relationships that may exist between the genetic variants and phenotypic characteristics to predict the outcome of interest. The AUC was used to compare model performance with differences in the AUC quantified using an approach described by DeLong et al. [38]. Additionally, we evaluated the sensitivity and specificity of each model. All the machine learning models were developed using R statistical software version 4.1.

## 3. Results

The characteristics of this study’s subjects are shown in Table 1. The training cohort in our analysis included 823 participants (323 cases, 500 controls), while the test cohort consisted of 548 participants (199 cases, 349 controls). The subjects in the training and test cohorts were 60% female and were from various stages across the life course (from early childhood to late adulthood). The mean ages were similar across the training and test cohorts. Individuals were overweight on average, and their BMI levels ranged from underweight to obese. Approximately one third of the participants experienced exacerbations while on ICS.

A total of 271 variants met a suggestive GWAS significance threshold of *p* < 1 × 10^−5^ for ICS response, and a secondary significance threshold of 1 × 10^−2^ for the clumped SNPs. The GWAS inflation was low (lambda = 1.03). The full list of genetic variants annotated to 132 genes is shown in Supplemental Table S1. A q-q plot of the GWAS is shown in Supplementary Figure S1, and a Manhattan plot is shown in Supplementary Figure S2.

**Figure 1:**
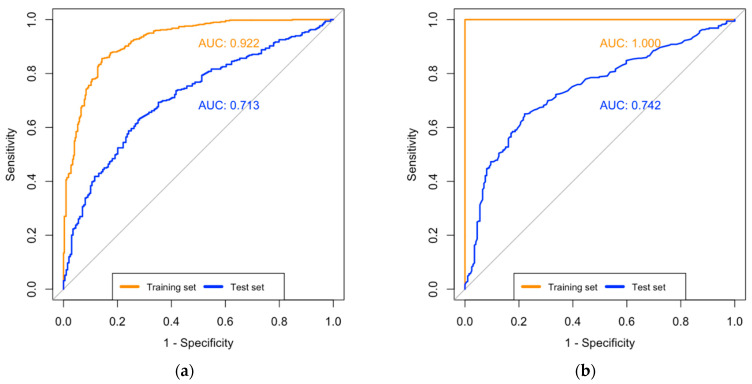

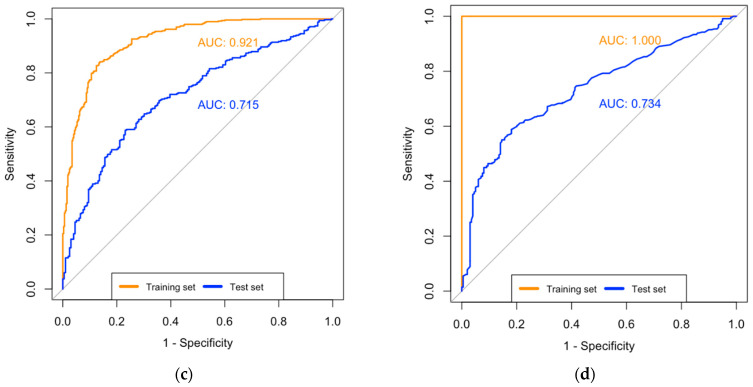
Model performance measured by the area under the receiver operating characteristic curve (AUC). (**a**) LASSO regression model including SNPs only. (**b**) LASSO regression model including SNPs and phenotype data. (**c**) Random forest model including SNPs only. (**d**) Random forest model including SNPs and phenotype data.

We used these sets of variants to train and fine-tune the machine learning models for predicting ICS response. The most optimal LASSO regression model employed a LASSO regularization term of 0.0187 and retained 89 of the 271 SNPs. The model achieved an AUC of 0.71 (95% CI 0.67–0.76) in the test cohort, with 57% sensitivity and 75% specificity (Table 2; Figure 1). The most optimal random forest model retained 270 SNPs, achieving an AUC of 0.74 (95% CI 0.70–0.78) in the test cohort, with 70% sensitivity and 68% specificity (Table 2; Figure 1). Table 3 summarizes the top most important variants identified by each model. Many of these variants are located near or within genes with known links to asthma and allergic disease phenotypes. These genes include those associated with corticosteroid responses in asthma (*TPSAB1, FBXL16*), asthma symptoms and severity (*ABCA7, CNN2, PTRN3,* and *BSG/CD147*), airway remodeling (*ELANE, FSTL3*), mucin production (*GAL3ST*), lipid peroxidation and pro-inflammatory leukotriene levels (*GPX4*), allergic asthma (*ZFPM1, SBNO2*), and others.

In addition to constructing predictive models based on SNPs alone, we also incorporated phenotypic data (sex, age, and BMI) into our predictive models. In the random forest model, all three phenotype variables were selected for inclusion. In the LASSO regression model, only BMI was selected for inclusion. In both models, the inclusion of both SNP data and these phenotypic variables did not show improved predictive accuracy over the models that included SNPs alone (Table 2).

Our study combined data from different sources and, thus, may be subject to batch effects (i.e., subgroups of measurements that have qualitatively different behavior across conditions and are unrelated to the biological or scientific variables of a study [39]). To address the potential confounding by batch effects, we applied a bootstrap resampling approach to train the predictive models. A qualitative assessment of the relationship between group membership and the first two principal components defining the association between SNPs and ICS response further found no evidence of correlation between the variables (Supplementary Figure S3), suggesting the absence of batch effects.

## 4. Discussion

Genome-wide association studies of response to asthma medications have identified multiple genetic variants, but few studies have combined the effects of these individual SNPs into a single pharmacogenetic model. In this work, we utilized data from multiple cohorts to train and test machine learning models for predicting responsiveness to ICS, the most common controller medication for asthma. We report three main findings. First, applying LASSO regression and random forest to the top GWAS hits allowed us to select the most relevant SNP contributors to ICS treatment response, with a relatively high predictive accuracy. Second, many of the SNPs selected by our models are located within the genes associated with corticosteroid response in asthma, asthma severity, and immune function, suggesting a strong underlying biological plausibility for our models. Third, the inclusion of genetic variants alone was sufficient to predict the response to ICS treatment. The addition of phenotypic information did not enhance the performance of our pharmacogenetic models.

The development of a polygenic model for asthma treatment response presents several challenges. The high dimensionality of the GWAS data and the correlation patterns between SNPs can hinder the process of SNP selection for the pharmacogenetic model. Polygenic prediction models are also prone to overfitting, which can falsely inflate prediction estimates. To address these issues, we applied two machine learning approaches that are well suited to performing prediction tasks using high-dimensional data. To optimize model development, we prioritized associations at a prespecified *p*-value threshold and accounted for correlation patterns by LD clumping and filtering. The number of SNPs selected for inclusion was further reduced by two-thirds by the LASSO regression modeling approach, while the random forest model retained all but one SNP that met the significance thresholds for GWAS and LD clumping. Although the random forest model outperformed the LASSO regression model (AUC of 0.74 vs. 0.71), the difference in the AUC did not reach statistical significance (*p* = 0.06). Given the small study sample, we were unable to draw any reliable conclusions about the relative effectiveness of the two algorithms. However, it is interesting to note that the LASSO regression modeling approach excluded specific variants previously shown to be associated with response to corticosteroids. For example, the SNPs in or near *FBXL16* and *TPSAB1* were retained in the random forest model but not in the LASSO regression model, both of which show differential gene expression in response to corticosteroids either in vitro or in vivo. Mostofa et al. reported that *FBXL16* is part of the “early responder” gene expression profile in human bronchial epithelial cells in individuals with asthma within 6 hours of treatment with budesonide, an ICS [40]. Mast cell *TPSAB1* expression is associated with a better clinical response to corticosteroids in individuals with asthma [41], and corticosteroids have been shown to suppress *TPSAB1* expression in bronchial epithelial cells [42].

Several other genes included in the models were not connected to the ICS response mechanisms per se, but have been linked to asthma severity, asthma control, airway remodeling, and Th2-mediated responses. *ABCA7* gene expression is associated with nocturnal asthma symptoms in individuals with a polymorphism in *NPSR1* [43]. *BSG* (also called *CD147*) is a potential target for asthma treatment therapy. Anti-CD147 treatment significantly reduces airway epithelial mucin production and bronchial hyperreactivity to methacholine challenge in murine models of asthma [44]. *CNN2* is associated with the development of severe asthma [45]. *PRTN3* encodes for an airway biomarker associated with neutrophil activation and poor asthma control [46]. *Piezo-1* regulates the function of tight junction proteins within the airway epithelial cells of individuals with asthma following mechanical stress that mimics bronchoconstriction [47]. *ELANE*, the gene for neutrophil elastase, is expressed in bronchial epithelial cells and may play a role in airway remodeling by contributing to smooth muscle hypertrophy [48]. *FSTL3* shows a reduced expression in the bronchial epithelium of individuals with asthma, which impairs the regulation of fibroblasts involved in remodeling [49]. *MIER2* is differentially expressed among obese compared with normal-weight asthmatic children [50]. *PTBP1* plays an important role in the humoral immune response [51] and *PTBP1* deletion in dendritic cells has been shown to enhance asthma exacerbation [52]. The genes associated with mucin production (*GAL3ST2*) [53], leukotriene synthesis (*GPX4*) [54], Th2-mediated allergic asthma (*PTBP1, ZFPM1, SBNO2,* and *EGFL7*) [52,55,56,57], and IgE mediated allergy (*PAK2*) [58] were also represented in our polygenic prediction models of ICS response.

Additionally, several of the top SNPs identified by our models are in/near genes that have been linked to the epigenetic mechanisms in asthma. *PRR25* is associated with utero smoke exposure (IUS) [59]. In individuals with asthma, a history of IUS exposure has been shown to reduce the efficacy of ICS for decreasing airway responsiveness [60]. *C1orf159*, *BTBD2,* and *HMHA1* are also associated with air pollution variables and lung function traits [61,62,63,64,65]. For example, the increased expression of *C1orf159* appears to exacerbate susceptibility to air pollution’s effect on pulmonary function, *BTBD2* is downregulated in the small airway epithelium in response to PM2.5 exposure, and *HMHA1* is associated with PM10 exposure and smoking. These findings highlight the need for more research on the role of environmental and epigenetic factors contributing to the response to asthma therapeutics.

It is interesting to note that the phenotypic characteristics (BMI, sex, and age) did not improve the predictive accuracy of our pharmacogenetic models of ICS response. While we did not have detailed asthma phenotypic information to add to our models, the SNP predictors in the genes described above suggest that a genetic predisposition towards particular asthma phenotypes (e.g.,Th2-mediated asthma) may be a key factor in predicting response to ICS.

Our study has several strengths. We used data from multiple cohorts, and across multiple age ranges to develop our polygenic risk prediction models. The models, composed of over 132 genetic variants across the genome, achieved a relatively high prediction accuracy for ICS treatment response in the test cohort. The inclusion of multiple cohorts, with participants across a wide range of ages, suggests that the predictive models are generalizable to pediatric as well as adult populations. However, the generalizability of our study is limited by the small sample size and the inclusion of Caucasian participants only. Future studies will be required to determine whether our polygenic prediction models perform equally well in other racial/ethnic populations. The limited sample size of our dataset also precluded sex-specific and age group-specific analyses. Additionally, we included BMI as a predictor, but BMI is an imperfect measure of adiposity, especially in children. The inclusion of richer phenotypic data has the potential to improve risk prediction. Furthermore, a predictive test with an AUC of 0.74 may not be appropriate for clinical use yet; however, this study demonstrates the promise of prediction models for ICS use. There is a further opportunity to improve the prediction by including rare variants that can only be detected through whole genome sequencing and are, therefore, not captured in our analysis.

In summary, we have developed machine learning prediction models to predict ICS response in asthma. These findings may ultimately inform decisions about ICS treatment in individuals with asthma.

## Figures and Tables

**Table 1:** Demographics of study population (n = 1371).

Variable	Training Cohort (n = 823)	Test Cohort (n = 548)
Sex		
Female, n (%)	500 (60.1)	331 (60.4)
Male, n (%)	323 (39.2)	217 (39.6)
Age, years, mean (SD)	25.5 (13.0)	25.9 (13.1)
BMI, kg/m^2^, mean (SD)	26.8 (8.3)	26.2 (7.5)
Exacerbation while on ICS, n (%)	323 (39.2)	199 (36.3)

**Table 2:** Performance of the most optimal prediction models.

Performance Measure	LASSO Regression Model		Random Forest Model	
	Training Cohort (n = 823)	Test Cohort (n = 548)	Training Cohort (n = 818)	Test Cohort (n = 546)
Models including SNPs only				
AUC	0.94 (0.93–0.96)	0.71 (0.67–0.76)	1.00 (1.00–1.00)	0.74 (0.70–0.78)
Sensitivity	0.87	0.57	1.00	0.70
Specificity	0.86	0.75	1.00	0.68
Models including SNPs and phenotype data				
AUC	0.92 (0.90–0.94)	0.71 (0.67–0.76) *	1.00 (1.00–1.00)	0.73 (0.69–0.78) ^
Sensitivity	0.87	0.59	1.00	0.69
Specificity	0.83	0.72	1.00	0.63

* AUC not statistically significant when compared to the model including SNPs only (*p* = 0.969). ^ AUC not statistically significant when compared to the model including SNPs only (*p* = 0.79).

**Table 3:** The top most important variables in model development. (a) LASSO regression model; (b) Random forest model.

Chr:Postion	Rs Number	Nearest Gene	Reference Allele	Effect Allele	Effect Allele Frequency	Variable Importance *
(a)						
21:20856221	rs4818452	*RPL37P4*/*TMPRSS15*	G	C	0.44	100
10:37228865	rs1852484	*ANKRD30A*	G	A	0.27	91.9
3:196520050	rs79390411	*PAK2*	C	T	0.13	76.8
4:77473026	rs114847105	*SHROOM3*	T	A	0.28	62.1
13:69770987	rs9541819	*KLHL1*	G	A	0.07	61.8
8:51961588	rs10093174	*PXDNL*	G	T	0.07	54.4
4:111218700	rs75800589	*ZBED1P1*	G	A	0.14	51.4
19:339675	rs878685	*MIER2*	C	T	0.39	49.9
11:6718704	rs1466977	*MRPL17*	T	G	0.29	48.7
2:215660643	rs6747962	*BARD1*	C	A	0.16	47.9
4:118248606	rs75774008	*TRAM1L1*	T	G	0.05	47.2
19:654968	rs4594371	*RNF126*	G	A	0.05	46.5
16:88731011	rs752843	*RNF166*	A	G	0.14	42.4
9:139502019	rs55892012	*EGFL7*	G	A	0.10	42.1
7:334719	rs36177169		C	T	0.09	39.1
17:18584142	rs116808485	*ZNF286B*	A	G	0.08	38.1
16:88826073	rs2278053	*PIEZO1*	G	C	0.31	35.1
16:88555879	rs34319485	*ZFPM1*	G	A	0.25	35.1
16:870711	rs2382764	*PRR25*	T	C	0.07	34.3
15:20587599	rs1846765	*GOLGA6L6*	G	C	0.12	33.1
19:2012477	rs4405674	*BTBD2*	T	G	0.36	29.1
22:17164773	rs361799	*TPTEP1*	C	T	0.05	28.9
1:1065296	rs4072537	*C1orf159*	T	C	0.25	28.4
16:1194047	rs4288998	*CACNA1H*	A	G	0.22	26.6
9:140304779	rs9414736	*EXD3*	A	G	0.27	26.6
8:51478714	rs17709272	*SNTG1*	G	T	0.37	26.2
17:80214198	rs12601586	*CSNK1D*	A	G	0.19	26.0
(b)						
19:1086211	rs1061233	*HMHA1*	G	A	0.31	100
21:20856221	rs4818452	*RPL37P4*/*TMPRSS15*	G	C	0.44	97.7
19:780209	rs7343137	*PTBP1*	T	C	0.38	95.3
1:1097291	rs61768478	*MIR200B*	C	A	0.17	95.2
19:840090	rs351109	*PRTN3*	T	C	0.34	94.5
19:710050	rs8109226	*PALM*	T	G	0.22	90.6
16:1184532	rs34056718	*CACNA1H*	C	T	0.39	90.3
19:1773999	rs4807140	*ONECUT3*	C	T	0.33	88.0
1:1053385	rs4970408	*C1orf159*	C	T	0.38	85.9
11:6718704	rs1466977	*MRPL17*	T	G	0.29	85.7
8:144987934	rs6999129	*MIR661/EPPK1*	A	T	0.37	85.4
10:37228865	rs1852484	*ANKRD30A*	G	A	0.27	84.9
19:1723463	rs10413694	*ONECUT3*	A	G	0.38	84.1
16:798229	rs8050465	*NARFL*	G	A	0.34	84.0
8:51478714	rs17709272	*SNTG1*	G	T	0.37	83.1
15:20303075	rs76044586		T	C	0.19	83.4
19:702286	rs8106722	*PALM*	G	C	0.24	83.3
1:949608	rs1921	*ISG15*	G	A	0.29	81.6
19:1063930	rs4807499	*ABCA7*	C	T	0.26	80.6
19:646891	rs10403235	*FGF22*	G	A	0.28	80.5
19:539279	rs2288956	*CDC34*	C	T	0.19	80.1
16:877334	rs28541981	*PRR25*	C	T	0.34	79.7
19:1766737	rs12978813	*ONECUT3*	C	A	0.26	79.6
16:32603025	rs28887512		A	G	0.41	79.2
3:196520050	rs79390411	*PAK2*	C	T	0.13	79.2
19:2012477	rs4405674	*BTBD2*	T	G	0.36	79.2
9:140304779	rs9414736	*EXD3*	A	G	0.27	78.9

* The variable importance is a ranked variable (ranging from 0 to 100) that quantifies the importance of each SNP in the prediction models for ICS response.

## Data Availability

The data underlying this article were provided by the following studies by permission: the Childhood Asthma Management Program (CAMP), the Childhood Asthma Research and Education (CARE) network, the Asthma Clinical Research Network (ACRN), and data from two biorepositories linked to deidentified electronic health records from the PharmacoGenomic discovery and replication in very large POPulations (PGPop) cohorts—the Marshfield Clinic Personalized Medicine Research Project (PMRP) and Vanderbilt University Medical Center’s BioVu program (BioVu). The data will be shared on request to the corresponding author with permission from the principal investigators of these studies.

## Supplementary Materials

The following supporting information can be downloaded at: https://www.mdpi.com/article/10.3390/jpm14030246/s1, Table S1: Description of study cohorts; Figure S1: Q-Q plot of GWAS p-values; Figure S2: Manhattan plot of GWAS; Figure S3: Principal components of data by study group.
