# Supplementary material for: Machine Learning Prediction of Treatment Response to Inhaled Corticosteroids in Asthma
Source: J Pers Med. 2024 Feb 25;14(3):246. doi: 10.3390/jpm14030246 (PMC10970828; doi:10.3390/jpm14030246)

Supplementary Materials

Supplementary Table S1. Description of study cohorts

Dataset	Study participants
Childhood Asthma Management Program (CAMP)	Children 5-12 years of age with mild-to-moderate persistent asthma
Pediatric Asthma Controller Trial (PACT)	children 6-14 years of age with mild-to-moderate physician-diagnosed asthma
Characterizing Response to Leukotriene Receptor Antagonist and Inhaled Corticosteroid (CLIC)	children 6-17 with mild-to-moderate asthma
Asthma Clinical Research Network (ACRN)	Adults 18 to 65 years with physician-diagnosed asthma
Marshfield Clinic Personalized Medicine Research Project (PMRP)	Adults aged 18 and above who resided in 1 of 19 zip codes surrounding Marshfield, WI
Vanderbilt University Medical Center's BioVu program (BioVU)	Patients at Vanderbilt University Medical Center

Supplementary Figure S1. Q-Q plot of GWAS p-values

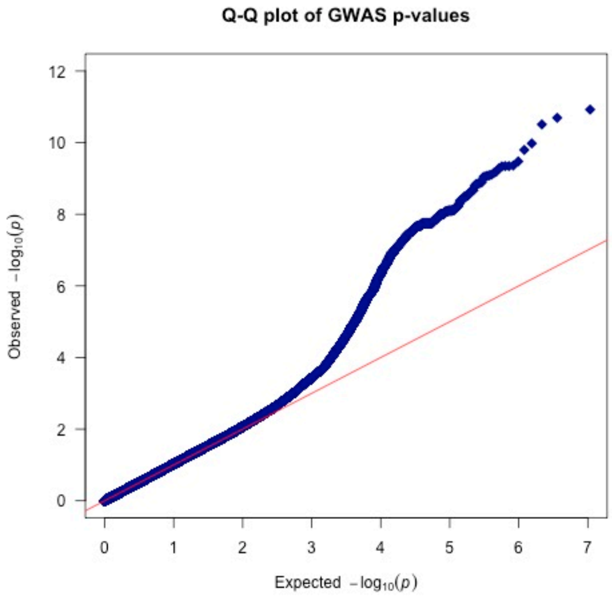

Supplementary Figure S2. Manhattan plot of GWAS

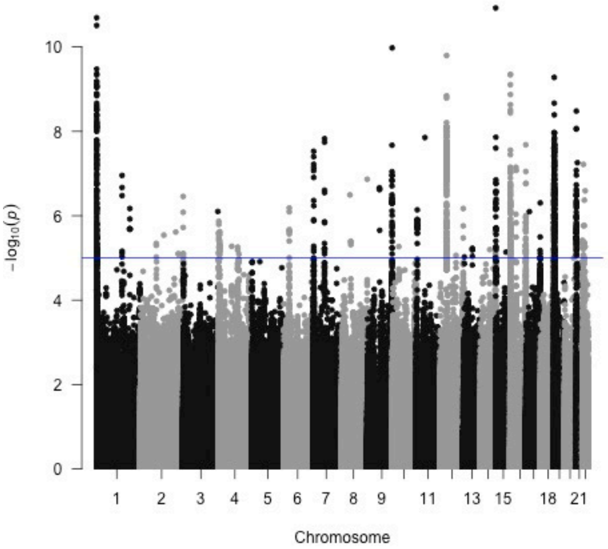

Supplementary Figure S3. Principal components of data by study group.

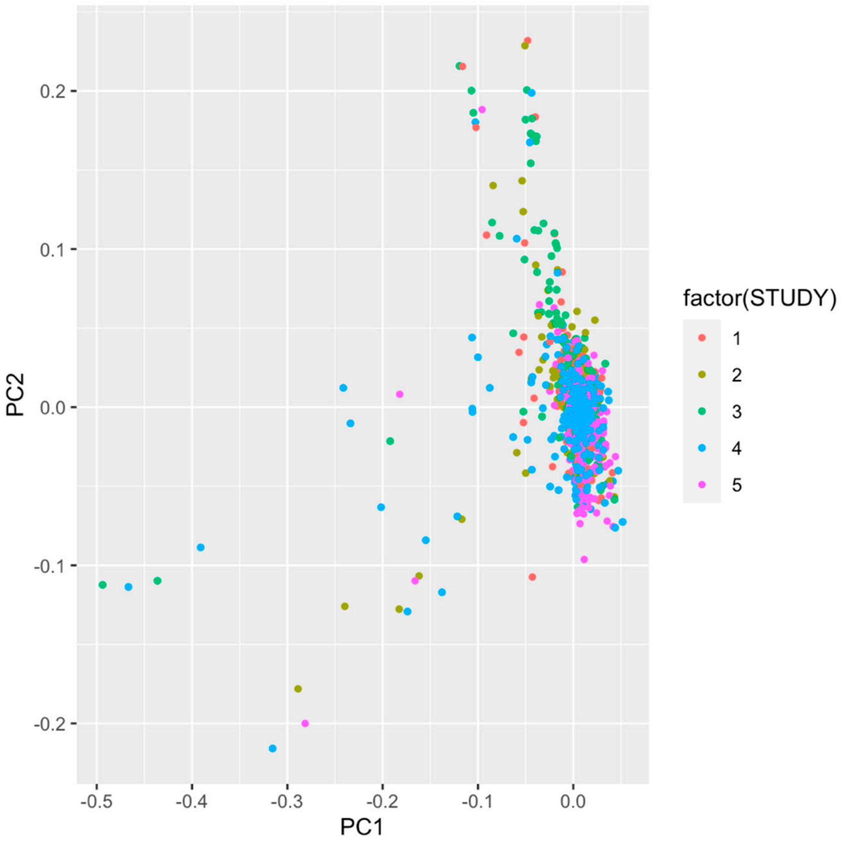

Supplement: Supplementary file 1 [file jpm-14-00246-s001.zip › jpm-2794626-supplementary.pdf]
